# Assessing the accessibility of care facilities for older adults with integration of their choice preferences: a case study of Fuzhou’s main urban area, China

**DOI:** 10.3389/fpubh.2025.1679888

**Published:** 2025-11-04

**Authors:** Qiuyi Zhang, Siying Wu, Gang Lin, Xiaofen Yan

**Affiliations:** ^1^College of Architecture and Urban Planning, Fujian University of Technology, Fuzhou, China; ^2^College of Design, Fujian University of Technology, Fuzhou, China; ^3^Zhangzhou Health Vocational College, Zhangzhou, China

**Keywords:** care facilities for older adults, spatial accessibility, improved Gaussian two-step floating catchment area method, Fuzhou’s main urban area, subjective willingness

## Abstract

Against the backdrop of rapid population aging in China, optimizing the spatial allocation of older adults’ care facilities has become increasingly important. This study evaluates both objective and preference-based accessibility to address gaps in precision planning for aged care. Focusing on Fuzhou’s main urban area, this research investigates three facility types: institutional care service facilities, community day care centers, and home-based care service stations. Using a modified Gaussian two-step floating catchment area (MG2SFCA) method with residential communities as analytical units, this research conduct accessibility analysis integrated with older adults’ choice preferences to quantify disparities between actual and willingness-weighted accessibility. Findings reveal significant spatial mismatches between facility distribution and older adults’ needs across all three types. Building on this, we develop street-level typological classifications (Gain/Attenuation/Risk) to propose context-specific optimization strategies. The study’s innovations lie in: (1) advancing analytical precision through micro-scale community units, and (2) incorporating subjective care preferences into spatial evaluation models, thereby providing evidence-based planning references for age-friendly city development.

## 1 Introduction

According to data released by the National Bureau of Statistics, by the end of 2024, the population aged 60 and above in China reached 310.31 million, exceeding 300 million for the first time, accounting for 22.0% of the national total population. With the shrinking of family sizes, the importance of social care for older adults has become increasingly prominent, and the demand for care facilities for older adults has grown rapidly (34). To implement the national strategy of actively responding to population aging, the “*14th Five-Year Plan for National Aging Cause Development and Older Adults’ Care Service System*” points out that street-level regional older adults’ care service centers and community older adults’ care service institutions should jointly build a 15-min home-based older adults’ care service circle (1). The “*Fujian Provincial Older Adults’ Care Service Regulations*” proposes to promote the integrated development of home-based, community, and institutional older adults’ care (2). These policies indicated that attention should be paid to the coordinated allocation of home-based, community, and institutional care service facilities, and a sound older adults’ care service facility system is an effective way to actively build an aging society in the field of planning. By the end of 2023, the number of older adults aged 60 and above in Fuzhou City reached 1.491 million, accounting for 21.66% of the city’s registered population, showing a severe situation of population aging (3).

Extensive research exists on the functional configuration of community older adults’ care facilities and service demand. Spatial accessibility evaluation effectively identifies public service facility shortages (4). The two-step mobile search method is a commonly used tool for measuring the accessibility of older adults care facilities (5, 6, 31). The core advantage of this method is that it can take into account the interaction between supply and demand, and it can visualize the accessibility results in the form of supply–demand ratio (7). In order to better fit the actual supply and demand situation of older adults’ service organizations, existing studies have expanded the method from several dimensions: for example, introducing a distance decay function to reflect the decreasing accessibility with increasing distance (8), incorporating a multi-level service radius to fit the service coverage of older adults’ service organizations of different sizes (9), and refining the spatial unit of analysis to improve the accuracy of the measurement (10).

Given that spatial accessibility is influenced by multiple factors—including supply/demand scale and distance—a modified Gaussian two-step floating catchment area (MG2SFCA) approach. By integrating a dynamic distance decay function, demand stratification weighting, and multi-modal transportation, it enhances resource supply–demand matching accuracy in complex urban environments. This approach addresses traditional models’ insufficient adaptability in heterogeneous regions (11). Consequently, this study employs MG2SFCA to analyze older adults’ care facility supply–demand relationships.

With regard to the accessibility of older adults’ service facilities, consideration was given to the size of the facilities (12, 13), spatial layout influencing factors (14, 33), as well as the study of the accessibility of older adults’ service facilities from the perspective of different modes of travel (15, 16). While existing research on care facilities for older adults has predominantly employed coarse spatial units such as districts or streets (17–19), these scales are often too large to accurately capture intra-regional accessibility disparities. Furthermore, analyses have primarily focused on objective, quantitative geographical assessments of accessibility (5, 20), largely neglecting the subjective preferences of older adults’ individuals when choosing facilities. To address these limitations, this study examines three tiers of older adults’ care services in Fuzhou’s main urban area, including institutional care (institutional care service facilities), community-based care (community day care centers), and home-based support (home-based care service stations). Utilizing MG2SFCA, this study evaluate spatial accessibility at the granular residential community level. Crucially, this assessment integrates the actual willingness of older adults to utilize care facilities. By comparing accessibility derived from purely spatial metrics with that incorporating user preference, the approach provides a dual-perspective analysis (subjective and objective) of real-world demand. This methodology aims to identify deficiencies in current facility layouts and offer actionable insights for future planning and research in older adults’ care provision.

## 2 Study area and data description

### 2.1 Study area: Fuzhou’s main urban area

**Figure 1:**
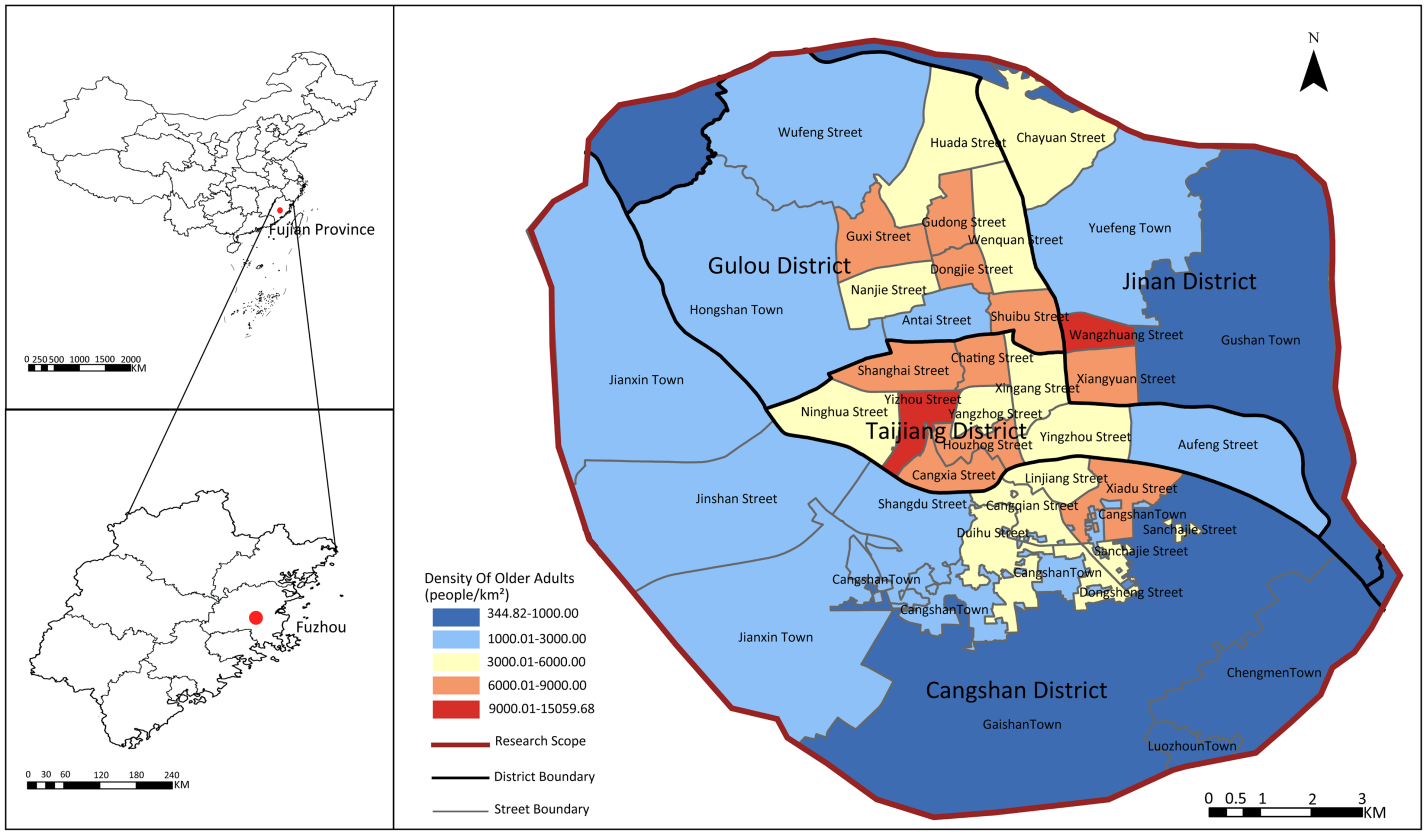
The study area.

The main urban area of Fuzhou refers to the area within the Third Ring Road, including Gulou District, Taijiang District, Cangshan District, and parts of Jin’an District, with a total research area of 174.21 km^2^ (see Figure 1). According to the data of the Seventh National Population Census, within the study area, Gulou District has 128,100 older adults, accounting for 26.19% of the total older adult population; Taijiang District has 82,400, accounting for 16.84%; Cangshan District has 125,100, accounting for 25.57%; and Jin’an District has 58,741, accounting for 12.01%. This paper takes the main urban area as the study area, with 2,275 residential communities as the research objects, focusing on the older adults in the residential communities.

The density of the older adult population in the main urban area shows a central-edge distribution: the central urban areas of Gulou District, Taijiang District, Jin’an District, and Cangshan District have a higher density of older adult population, while the outer urban areas have a relatively lower density. This area has a well-developed urban environment and a high degree of completeness of care facilities for older adults, but the contradiction between supply and demand still exists, so the selection of Fuzhou’s main urban area is representative.

### 2.2 Data description

#### 2.2.1 Older adults’ care service facility data

This paper integrates public data from the Fuzhou Older Adults’Care Service Network^1^ and the National Older Adults’ Care Service Information Platform,^2^ and extracts attribute information such as the name, longitude and latitude, institutional area, and number of beds of institutional care service facilities, community day care centers, and home-based care service stations using Python tools. In terms of setting service radius, the standards for community day care centers are clearly defined in relevant policy documents, while the service radius of institutional care service facilities and home-based care service stations is set differently according to their functional positioning (see Table 1 for details).

**Table 1:** Classification of care facilities for older adults research.

Facility type	Positioning and functions	Usage mode	Quantity (units)	Total number of beds (units)	Service radius (m)
Institutional care service facilities	Provide older adults people with all—inclusive services such as daily care, catering services, personal care, health care and rehabilitation, cultural and entertainment, psychological support, and end – of—life care. Full—care type care facilities for older adults. Including care homes and care hospitals, etc.		Gulou District (4)	436	1,500 ①②
		Long—term residence	Cangshan District (10)	4,191	
			Taijiang District (2)	535	
			Jian District (4)	355	
Community day care centers	Special facilities for older adults people to rest during the day, receive daily care services and other service projects.	Long—term residence or day return	Gulou District (11)	437	1,000③④
			Taijiang District (8)	314	
			Cangshan District (10)	242	
			Jinan District (4)	103	
Home—based care service stations	Provide industry management, resource connection, personnel training and other functions for home—based care services in urban and rural communities. Including county—level older adults care service guidance centers, sub—district older adults care service centers, town and township older adults care service guidance centers, community older adults care service centers, and village—level older adults care service stations. Among them, sub—district older adults care service centers and town and township older adults care service guidance centers are additionally set up with older adults care service functions.	Round—trip	Gulou District (68)	Mainly provide service assistance, no beds set; service radius is 1	500 ⑤⑥
			Cangshan District (92)		
			Taijiang District (52)		
			Jian District (64)		

① in the table refers to the “Standard for Planning of Urban Public Service Facilities” (GB50M2–2018); ② refers to the “Technical Guidelines for Community Life Circle Planning” (TD/T 1062–2021); ③ refers to the “Construction Standard for Community Day Care Centers for Older Adults” (JGJ143–2010); ④ is set according to the “Shanghai Special Plan for the Layout of Elderly Care Facilities (2013–2020),” which stipulates that the service radius of community older adults care facilities shall not exceed 1,000 m; ⑤ Code for Planning of Urban Public Facilities (GB 50442–2008); ⑥ Fujian Provincial Guidelines for the Planning and Allocation of Urban and Rural care facilities for older adults (Trial).

**Figure 2:**
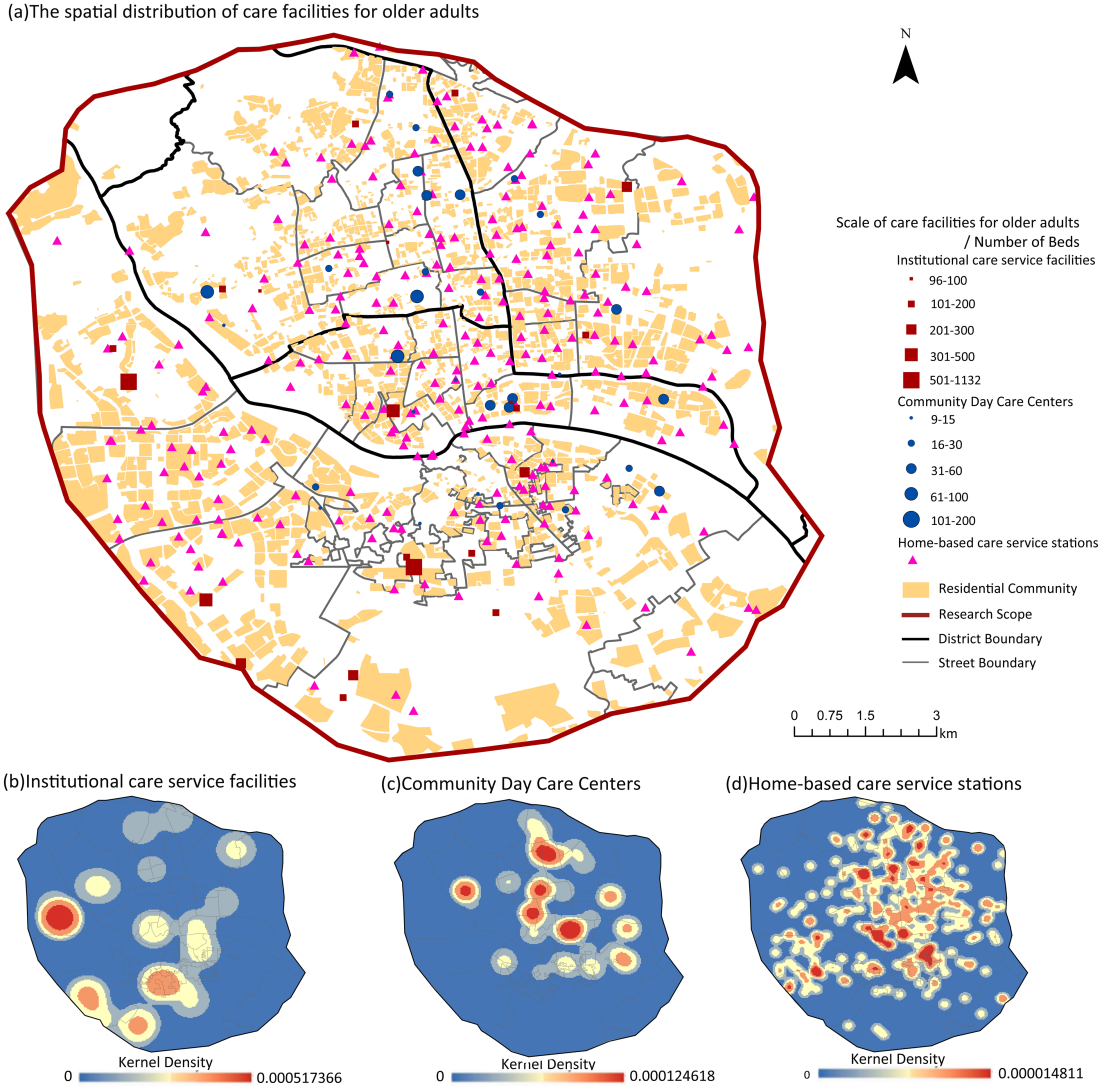
The spatial distribution of older adults’ care service facilities.

Based on their service functions and positioning, care facilities for older adults in the study area are categorized into three types. Institutional care service facilities include nursing homes and older adults’ care centers, which are bed-based and offer permanent living facilities. In the study area, there are 20 such facilities: 4 in Gulou District, 10 in Cangshan District, 2 in Taijiang District, and 4 in Jin’an District, with a total of 5,517 beds. Community day care centers are dedicated to providing daytime rest and daily care services for older adults offering both permanent living and daily round-trip options with bed facilities. There are 33 such centers in the study area:11 in Gulou District, 8 in Taijiang District, 10 in Cangshan District, and 4 in Jin’an District, with a total of 1,348 beds. Home-based care service stations include street (township) older adults’ care service centers and community (village) older adults’ care service stations, which focus on service functions without bed facilities. There are 276 such stations in the study area: 68 in Gulou District, 92 in Cangshan District, 52 in Taijiang District, and 64 in Jin’an District (see Figure 2).

#### 2.2.2 Residential community and older adults population data

**Figure 3:**
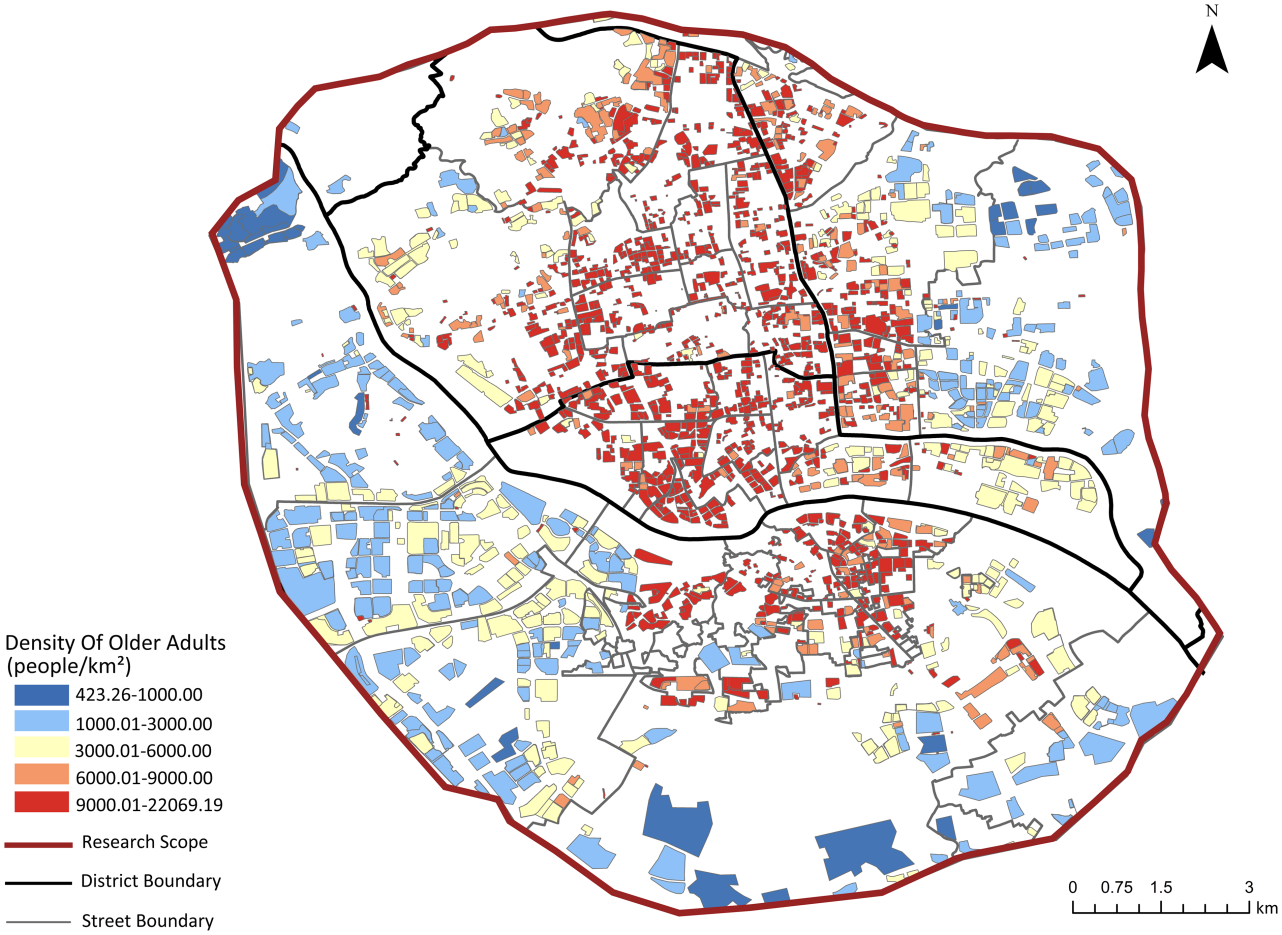
Calibrated spatial distribution of older adults population in residential communities.

Age-stratified population estimates were derived from 100 m-resolution WorldPop raster data.^3^ Using Google Earth Engine’s spatial analysis platform,^4^ this study implemented dasymetric mapping to disaggregate population counts to residential community units. While the 100 m resolution may not capture micro-scale variations within individual communities, this spatial granularity is well-suited for analyzing accessibility patterns at the residential community level (21, 22). The total older adults population (aged 60 and above) per community was calculated by summing relevant age cohorts (see Figure 3). These estimates were subsequently calibrated against street-level permanent resident data for the older adults population from Fuzhou’s Seventh National Population Census.

#### 2.2.3 Questionnaire survey data

A questionnaire survey was conducted between April 2024 and April 2025 in the four main urban districts of Fuzhou (Cangshan, Gulou, Taijiang, and Jin’an) to assess the older adults’ preferences regarding three types of older adults care modes. These preference data were subsequently used as weights for calculating the comprehensive spatial accessibility of care facilities for older adults.

The survey adopted a quota sampling method based on administrative districts to ensure basic spatial representativeness (23). Using data from Fuzhou’s Seventh National Population Census, the total sample of 1,034 valid questionnaires was proportionally allocated to the four districts according to the distribution of the population aged 60 and above. Within each district, convenience sampling was employed, with surveys conducted at locations frequently visited by older adults, such as community neighborhood committees, senior activity centers, parks, and plazas, until the predefined quota for each district was fulfilled.

A total of 1,034 questionnaires were distributed, and 997 valid responses were collected. The questionnaire included items on the preferred older adults care mode, age group, and residential community (see Table 2). The results indicated that 658 respondents (66%) preferred home-based care service stations, 219 (22%) opted for community day care centers, and 120 (12%) chose institutional care service facilities.

**Table 2:** Survey questionnaire on preferences for older adults care modes.

Questions	
1. Which older adults care mode do you prefer more?	A. Institutional Care Service Facilities (living in a professional nursing home or older adults care institution) B. Community Day Care Centers (living in the older adults care service center within the community) C. Home—based Care Service Stations (supported by family or the community)
2. What is your age group?	A. 60–70 years old B. 70–80 years old C. Over 80 years old
3. In which district of Fuzhou do you currently live?	A. Gulou District B. Taijiang District C. Cangshan District D. Jinan District E. Others

## 3 Research methodology

### 3.1 Facility-specific accessibility using MG2SFCA

The assessment of facility-specific accessibility in this study is based on several key assumptions. First, accessibility is modeled using Euclidean distance as a proxy for travel cost, that simplifies actual travel paths but provides a consistent and replicable metric for a large-scale urban analysis (24). Second, the model assumes walking as the primary travel mode, with thresholds set accordingly. This choice is deliberate, as it aligns with the policy goal of creating walkable “15-min life circles” and establishes a conservative baseline that highlights the situation for those most dependent on proximate services, such as older adults with limited mobility. Third, the older adults population is treated as a single group in terms of travel ability within the model. It does not differentiate between subgroups with varying physical capabilities. This simplification is necessary for a macro-level spatial equity analysis but means the results represent an average potential accessibility. These foundational assumptions allow for a clear and focused evaluation of the spatial distribution of facilities relative to the older adults population, isolating this factor from the complexities of multi-modal transportation networks or individual mobility differences, which are important avenues for future research.

The two-step floating catchment area method sets a threshold with a limited distance or time as the search radius, conducts two floating searches based on supply locations and demand locations respectively, and analyzes the supply capacity and actual demand of service facilities (25). Accessibility ( $\beta_{i}$ ) is evaluated using the MG2SFCA model on the same walking time scale. The MG2SFCA model is an integration of the widely used modified two-step floating catchment area (M2SFCA) model and the continuous Gaussian function for weighted distance decay, providing an estimate of spatial accessibility (26). In this paper, based on the original two-step floating catchment area method, the distance decay function is considered to improve the accuracy of evaluating the accessibility of older adults’ care service facilities in Fuzhou. The formula for calculating spatial accessibility based on MG2SFCA is:


$$\beta_{i}=\sum_{j,j\in [d_{\mathrm{ij}}\leq d0]}\frac{G_{j}f(d_{\mathrm{ij}})f(d_{\mathrm{ij}})}{\sum_{k,k\in [d_{\mathrm{jk}}\leq d0]}P_{k}f(d_{\mathrm{kj}})}$$

Where β_i_ is the accessibility measure of residential community i; G_j_ is the supply capacity of the j-th older adults care institution; P_k_ is the number of older adults aged 60 and above in residential community k; d_ij_ is the distance from residential community i to older adults care institution j; d_jk_ is the distance from older adults care institution j to residential community k; d_0_ is the maximum distance threshold considering accessibility; f(d_ij_) is a continuous Gaussian function used to assign weights to distance d_ij_. The formula for the Gaussian distance decay function is:


$$f(d_{\mathrm{ij}})=\{\begin{array}{cc} \frac{e^{-\frac{1}{2}{\left(\frac{d_{\mathrm{ij}}}{d_{0}}\right)}^{2}}-e^{-\frac{1}{2}}}{1-e^{-\frac{1}{2}}} & \text{if}d_{\mathrm{ij}}\leq d_{0}\\ 0 & \text{if}d_{\mathrm{ij}}>d_{0} \end{array}$$

The function f(d_ij_) decreases as d_ij_ increases, meaning that the impact of care facilities for older adults on residential communities weakens with increasing distance. The accessibility indicators are standardized for comparative analysis, investigation at different spatial scales, and disparity calculation. According to the functional characteristics and service radius disparities of different types of care facilities for older adults, different distance thresholds are set for accessibility research: the distance threshold for older adults care institutions is 1,500 m on foot, 1,000 m on foot for community care centers, and 500 m on foot for home-based care service stations.

### 3.2 Comprehensive and willingness-weighted accessibility

Comprehensive spatial accessibility considers the three types of service facilities comprehensively to calculate the overall accessibility of care facilities for older adults. Assuming that the three types of facilities are equally important (each accounting for 1/3), the calculation of comprehensive spatial accessibility is:


α=33.33%×C1+33.33%×C2+33.33%×C3

willingness-weighted accessibility is calculated by assigning weights to different institutional facilities through questionnaire surveys. The willingness to choose home-based care service stations accounts for 66%, community day care centers for 22%, and institutional care service facilities for 12%. The final willingness-weighted accessibility of older adults care facilities is calculated as:


β=12%×C1+22%×C2+66%×C3

where C1, C2, and C3 are the spatial accessibility of institutional care service facilities, community day care centers, and home-based care service stations, respectively (13).

The disparity between willingness-weighted accessibility and comprehensive accessibility can reveal the matching degree between the supply of care facilities for older adults and actual demand, and the disparity is calculated as (27):


δ=β−α

Where δ is the disparity between standardized willingness-weighted accessibility and standardized comprehensive accessibility. To eliminate the impact of random fluctuations, a threshold range of −0.12 to 0.12 is set to judge the significance of the disparity. The threshold was determined based on the quartile distribution of the δ values, where the 25th and 75th percentiles were approximately −0.137 and 0.104, respectively (32). Thus, the ±0.12 range effectively captures the middle 50% of the data as reflecting no substantive disparity. When δ >0.12, it indicates that willingness accessibility is significantly higher than comprehensive accessibility, which is mainly due to the increase in the weight of home-based care service stations to 66% leading to enhanced regional accessibility, indicating that the spatial layout of facilities better meets the preference needs of older adults. If the δ value is in the range of 0.12 to −0.12, it indicates that willingness accessibility is basically consistent with comprehensive accessibility, and the weight adjustment has no substantial impact on facility accessibility. When δ <−0.12, it shows that willingness accessibility is significantly lower than comprehensive accessibility, meaning that the spatial allocation of care facilities for older adults does not match the actual demand, especially reflecting the insufficient supply of home-based care service stations.

### 3.3 Spatial pattern analysis: spatial autocorrelation

To thoroughly investigate the spatial distribution patterns and aggregation characteristics of accessibility to older adults care facilities, this study employs spatial autocorrelation analysis to systematically assess the spatial dependence and heterogeneity of accessibility values. Spatial autocorrelation analysis includes both global and local measures, effectively identifying interdependencies between spatial units (28).

The Global Moran’s *I* index is used to measure the overall spatial clustering degree of older adults care facility accessibility across the entire study area. Its calculation formula is as follows:


$$I=\frac{n}{\sum_{i=1}^{n}\sum_{j=1}^{n}w_{\mathrm{ij}}}\cdot \frac{\sum_{i=1}^{n}\sum_{j=1}^{n}w_{\mathrm{ij}}(x_{i}-\bar{x})(x_{j}-\bar{x})}{\sum_{i=1}^{n}{\left(x_{i}-\bar{x}\right)}^{2}}$$

Where *I* is the Global Moran’s *I* index, *n* is the total number of spatial units (2,275 residential communities in this study), *x*_*i*_ and *x*_*j*_ are the standardized accessibility values of the *i*th and *j*th residential communities respectively, *x̄* is the mean accessibility value of all communities, *w_ij_* is the element of the spatial weight matrix denoting the spatial adjacency relationship between communities *i* and *j*.

The value of Moran’s *I* ranges from [−1, 1]. An I > 0 indicates positive spatial autocorrelation, meaning high or low values tend to cluster. *I* < 0 indicates negative spatial autocorrelation, meaning high and low values are interspersed. And I ≈ 0 suggests a random spatial distribution. The significance of spatial autocorrelation is determined using a Z-test (*p* < 0.05).

The Local Moran’s I index is used to identify local spatial clustering patterns and outliers. Its calculation formula is:


$$I_{i}=\frac{\left(x_{i}-\bar{x}\right)}{\sum_{i=1}^{n}{\left(x_{i}-\bar{x}\right)}^{2}/(n-1)}\cdot \sum_{j=1}^{n}w_{\mathrm{ij}}(x_{j}-\bar{x})$$

Where *I_i_*​ is the Local Moran’s I index for the *i*th residential community, and the other variables are defined as above.

The LISA (Local Indicators of Spatial Association) analysis, based on the Local Moran’s I, classifies residential communities into five types of spatial association patterns: High-High cluster (HH), Low-Low cluster (LL), High-Low outlier (HL), Low-High outlier (LH), and Not Significant.

### 3.4 Statistical validation

The robustness of the preference weights used to calculate willingness-weighted accessibility (*β*) was assessed prior to analyzing accessibility disparities. Chi-square tests of independence examined whether preferences for older adults care modes varied across age groups (60–70, 70–80, >80 years) and administrative districts (Gulou, Taijiang, Cangshan, Jin’an) (30). The results indicated no significant association between age group (*X*^2^ = 4.35, *p* = 0.360) or district (*X*^2^ = 10.32, *p* = 0.112) and preference, supporting the use of a unified weighting scheme (66, 22, 12%) for the study population.

The Mann–Whitney U test was applied to quantitatively assess the disparity between comprehensive accessibility (*α*) and willingness-weighted accessibility (*β*) (35). This non-parametric test was selected because the accessibility values for each residential community violated the assumption of a normal distribution (as confirmed by a Shapiro–Wilk test, *p* < 0.05), thus precluding the use of parametric tests such as the paired t-test. The test was performed using the paired α and β values from all 2,275 residential communities. The null hypothesis (H₀) posited that the distributions of α and β were identical. A significant *p*-value (*p* < 0.05) would lead to the rejection of H₀, indicating a statistically significant difference between the two accessibility measures.

## 4 Spatial accessibility analysis of care facilities for older adults in Fuzhou’s main urban area

### 4.1 Spatial patterns of older adults’ care facility accessibility

**Figure 4:**
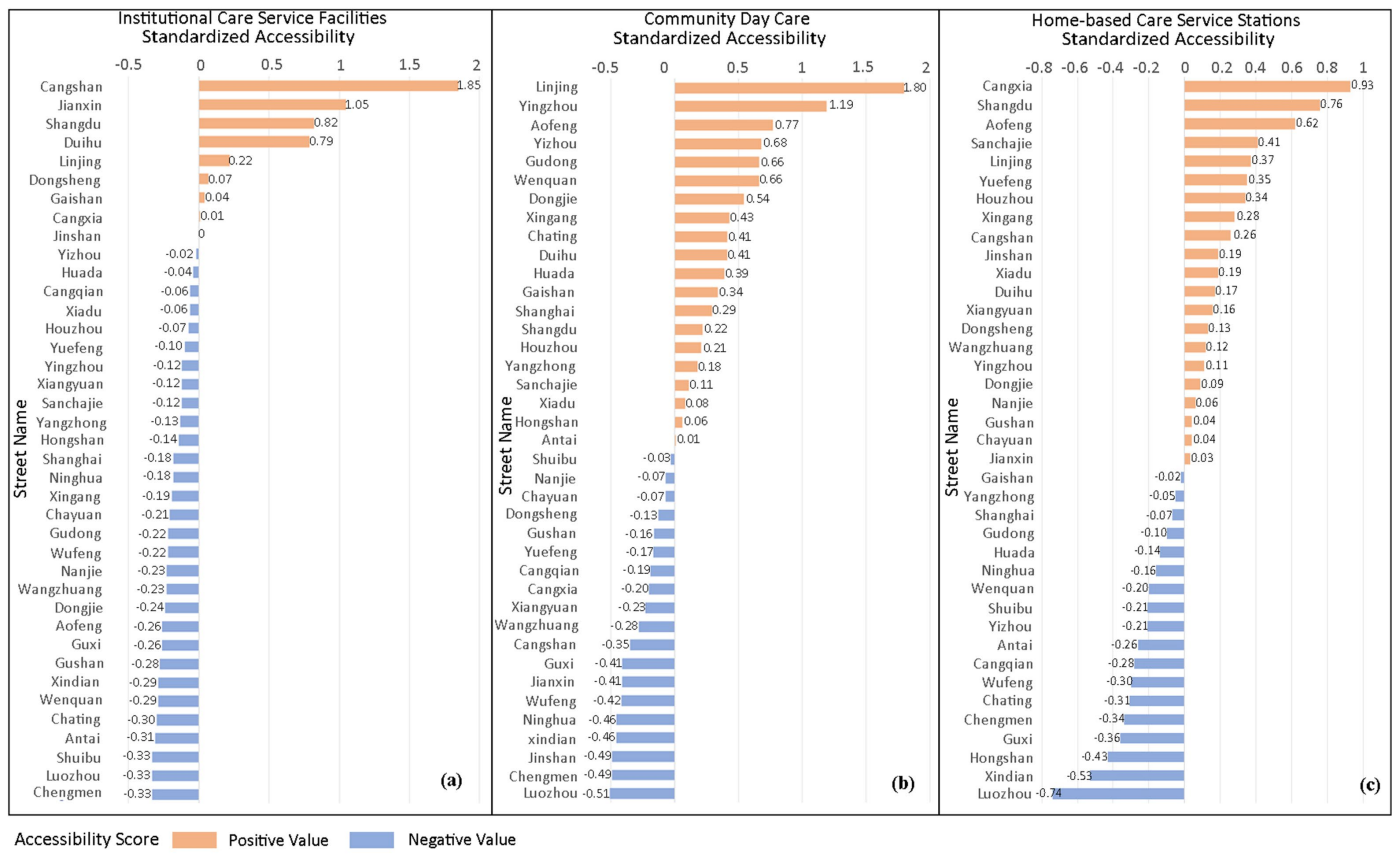
Street-level standardized accessibility (βi) for three older adults care facility types in Fuzhou main urban area.

The study uses the MG2SFCA to analyze the accessibility of care facilities for older adults, and the accessibility results are standardized (βi) to obtain five accessibility intervals (Figure 4): areas with “less than −0.3” indicate accessibility significantly lower than the average level; areas with “−0.3 to −0.1” indicate accessibility slightly lower than the average level; these two areas are identified as having insufficient care facilities for older adults; “−0.1 to 0.1” indicates accessibility close to the overall level; “0.1–0.3” indicates accessibility slightly higher than the average level; these two areas indicate reasonable facility layout; areas with “greater than 0.3” indicate accessibility significantly higher than the average level, indicating good planning and allocation.

#### 4.1.1 Institutional care facilities

**Figure 5:**
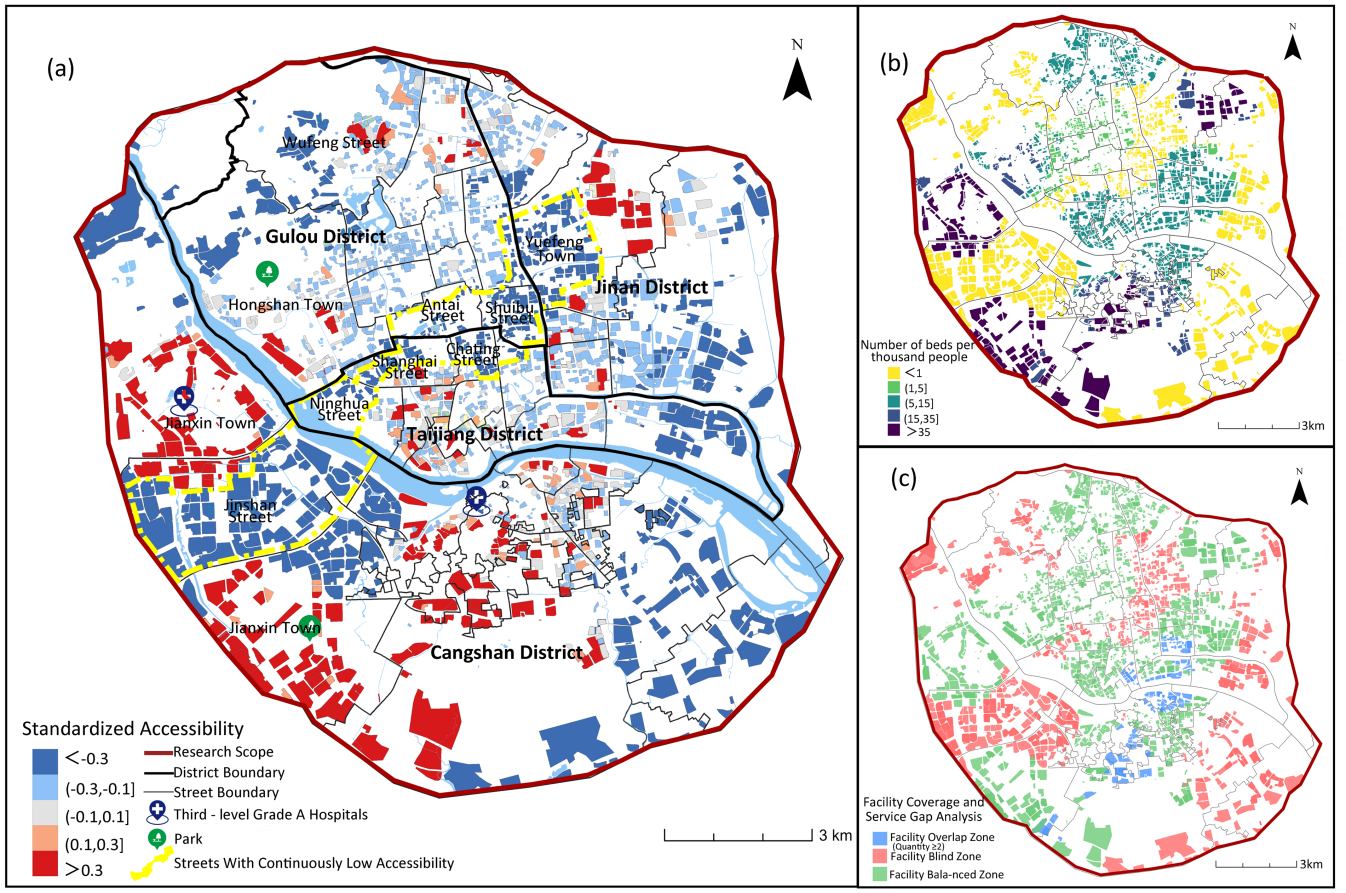
Spatial accessibility of institutional older adults care facilities.

Figure 5 reveals a core-periphery divergence in institutional care service facilities accessibility across Fuzhou’s main urban area, characterized by overall low service coverage. Higher accessibility is observed in outer urban zones, particularly in Jianxin Town (Cangshan District), Hongshan and Wufeng Streets (Gulou District), northern Yuefeng Town (Jinan District), and Minjiang riverside corridors. These areas exhibit bed-to-older adults ratios exceeding 40/1,000, indicating adequate capacity and are frequently clustered near hospitals or parks. Conversely, the urban core forms a contiguous low-accessibility belt extending southeast to southwest. This corridor encompasses the streets of Shuibu (accessibility index: −0.33), Chating (−0.30), Ninghua (−0.18), and Jinshan (0), as demarcated in Figure 4. These critically underserved areas exhibit bed-to-older adults ratios below 15/1,000, reflecting severe spatial inequity in institutional care provision.

Moreover, Figure 5c highlights significant spatial imbalances. 32.8% of residential communities lack institutional care service facilities, while 6.8% experience service oversaturation. These over-concentrated resources are primarily clustered along the Cangshan and Taijiang district boundary. This spatial mismatch originates from central urban land scarcity, where limited developable land creates planning deficits in bed capacity. Consequently, unmet demand in the urban core must be balanced through peripheral facility development, exacerbating intra-urban accessibility disparities.

**Figure 6:**
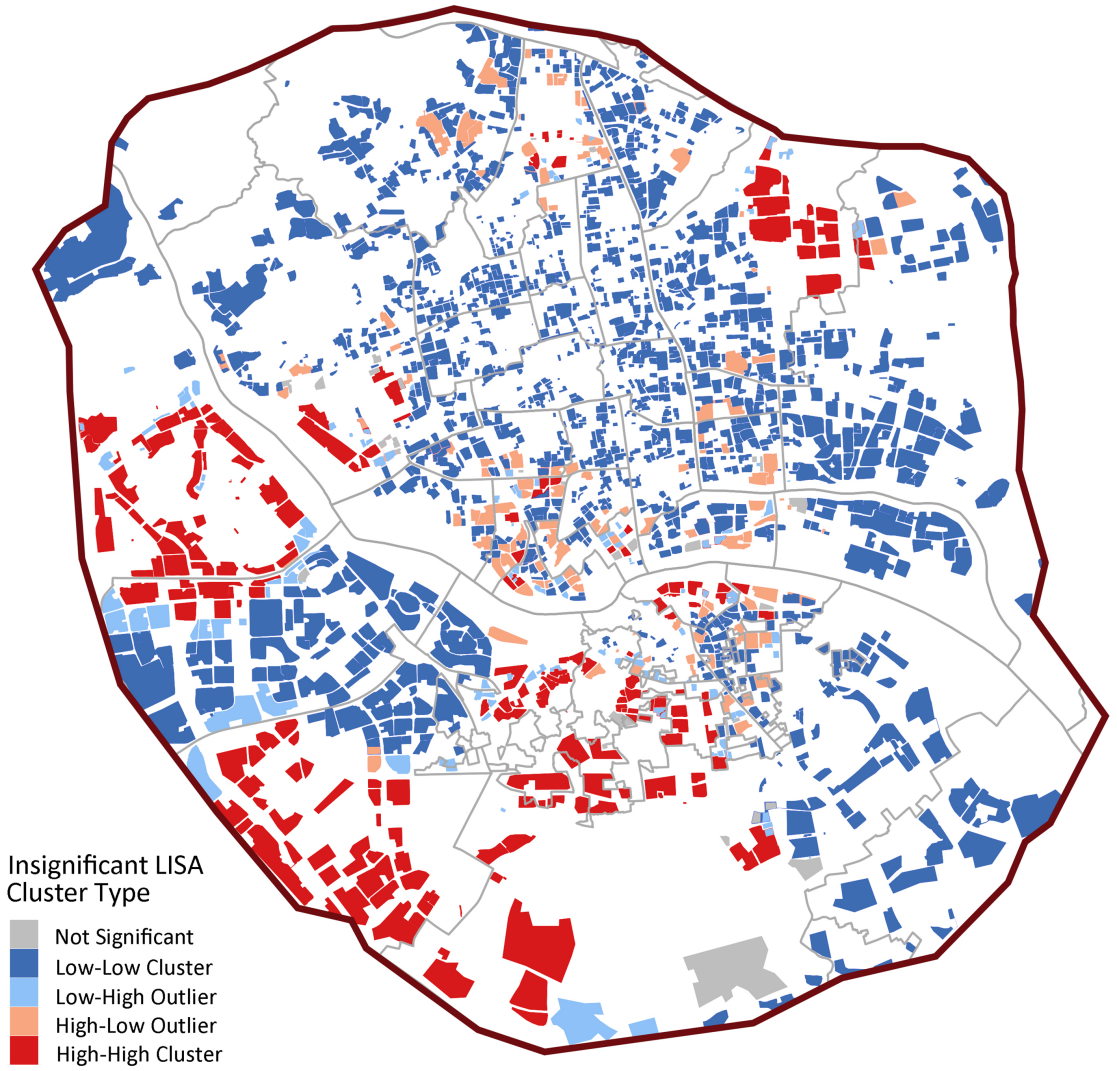
LISA cluster map of institutional care service facilities.

Spatial autocorrelation analysis confirms these patterns (Figure 6), showing significant positive spatial autocorrelation (Global Moran’s *I* = 0.3806, *p* < 0.001). LISA analysis indicates strong spatial polarization, with 74.64% of communities (1,698) forming large-scale “low-low” clusters primarily in peripheral and some central urban areas. Only 11.91% of communities (271) are “high-high” clusters, with a mean accessibility value (13.4) significantly higher than other types but substantial internal variation (SD = 15.8), reflecting extreme concentration of quality institutional care resources.

#### 4.1.2 Community day care centers

**Figure 7:**
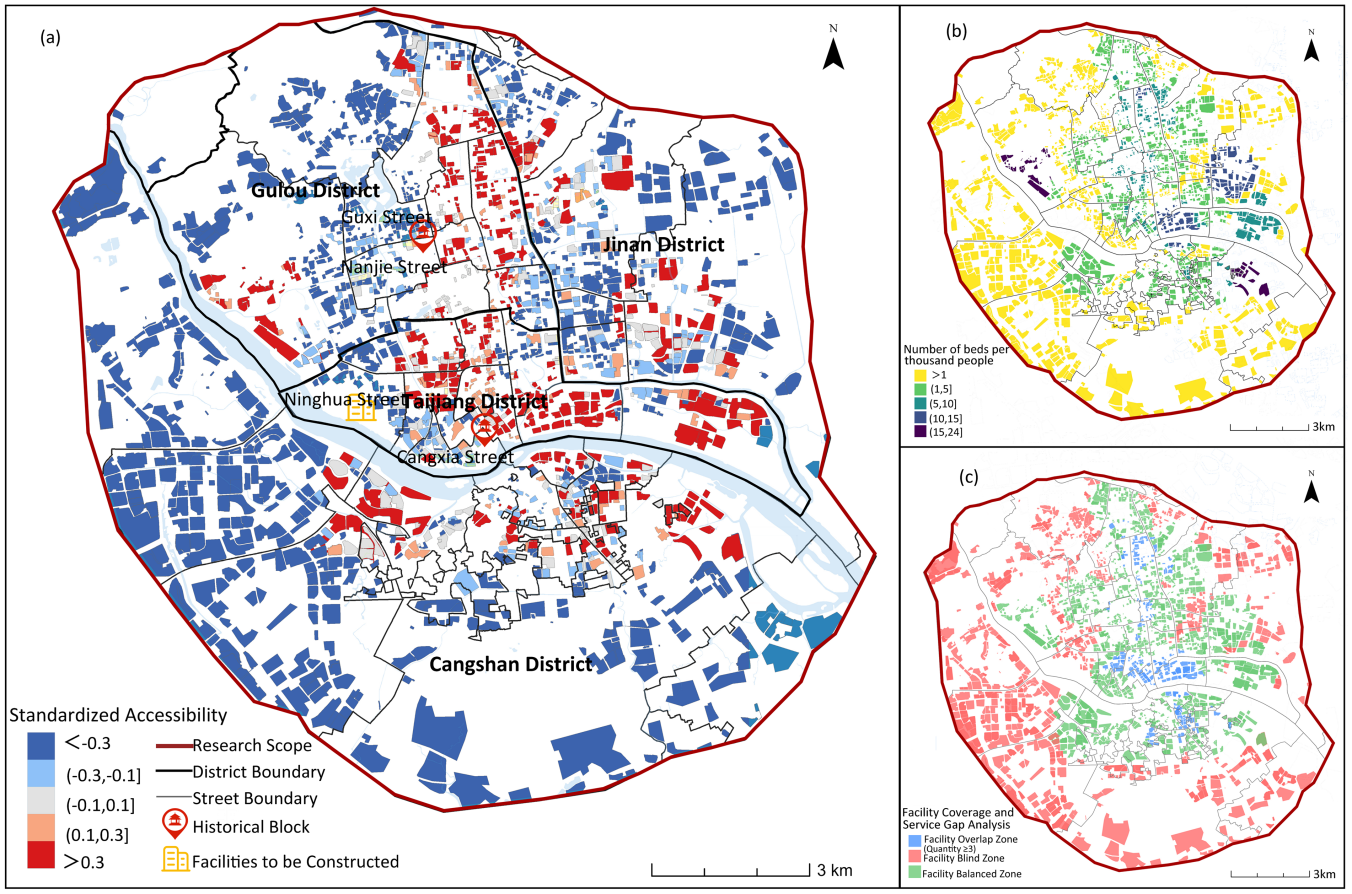
Spatial accessibility of community day care centers.

Figure 7 demonstrates an obvious central agglomeration pattern in community day care center accessibility across Fuzhou’s main urban area. High-accessibility zones concentrate in Gulou District, Taijiang District, and Minjiang riverside areas of Cangshan District, all central urban zones with bed-to-older adults ratios of 1–24 per 1,000. Conversely, peripheral areas in Cangshan and Jin’an Districts exhibit critically low accessibility (<1 bed/1,000 older adults), rendering services virtually inaccessible. While the urban core maintains overall advantage, localized deficits persist in the streets of Guxi (−0.41), Nanjie (−0.07), Ninghua Street (−0.46), Cangxia (−0.20) due to adjacency to historical blocks or undeveloped parklands. Notably, mountainous terrain and parkland restrictions exacerbate peripheral shortages, yet Cangshan’s non-riverside urban zones also show systemic accessibility failures, revealing significant facility undersupply beyond geographical constraints.

The study (see Figure 7c) indicates that 37.6% of residential communities lack access to community day care centers, while 11.8% exhibit service duplication in older adults care facility allocation. This demonstrates issues of resource over-concentration and inefficient distribution. These oversaturated service areas are primarily concentrated in core urban zones, where despite relatively good accessibility to community day care centers, supply exceeds actual demand.

**Figure 8:**
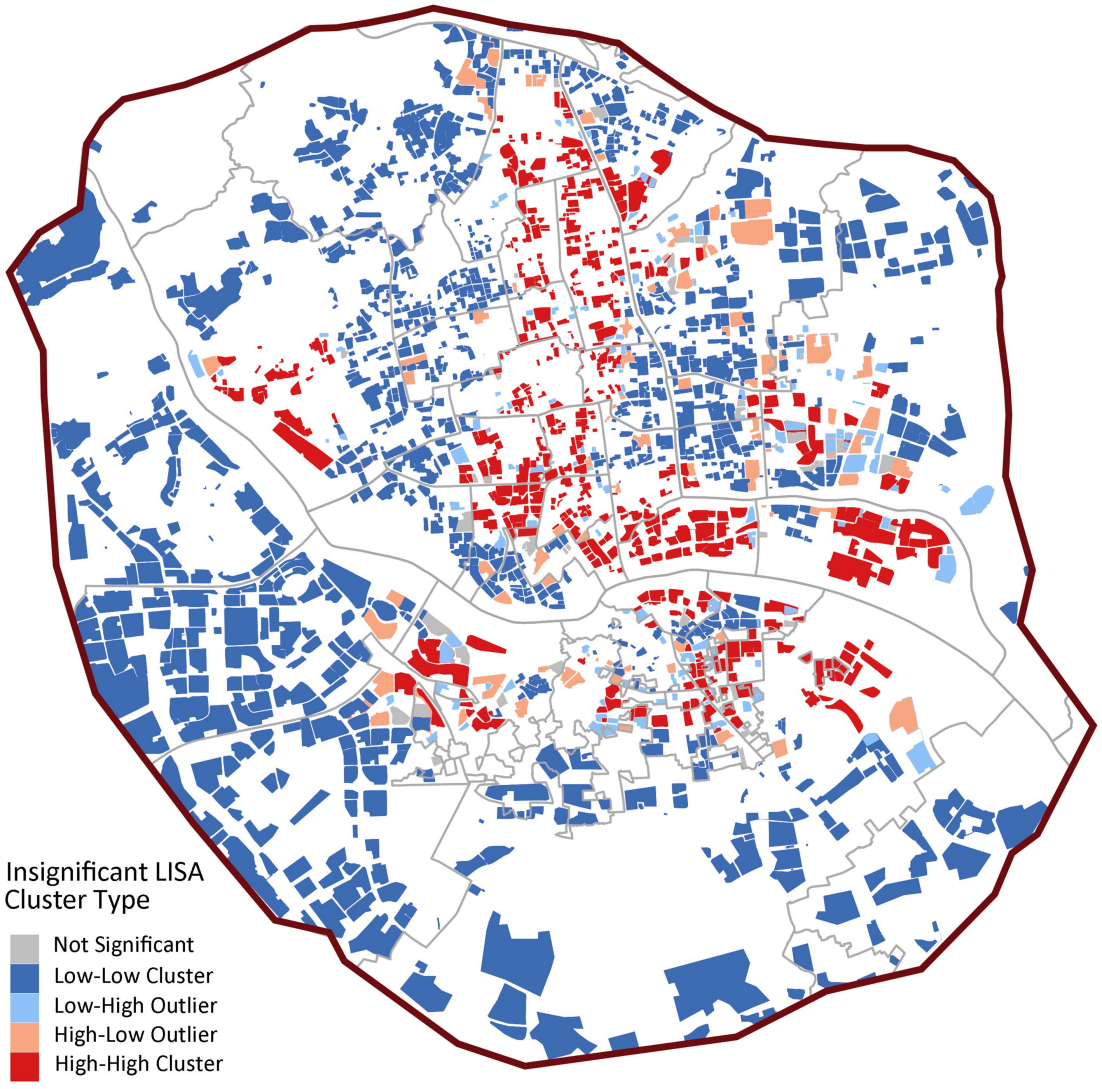
LISA cluster map of community day care centers.

In Figure 8, the spatial clustering is more pronounced (Global Moran’s *I* = 0.3842, *p* < 0.001), with 25.71% of communities (585) as “high-high” clusters, significantly higher than institutional facilities. These high-value clusters concentrate in central urban districts like Gulou and Taijiang, forming distinct service cores. However, 59.65% of communities (1,357) remain “low-low” clusters, confirming significant spatial coverage gaps (Figure 8).

#### 4.1.3 Home-based service stations

**Figure 9:**
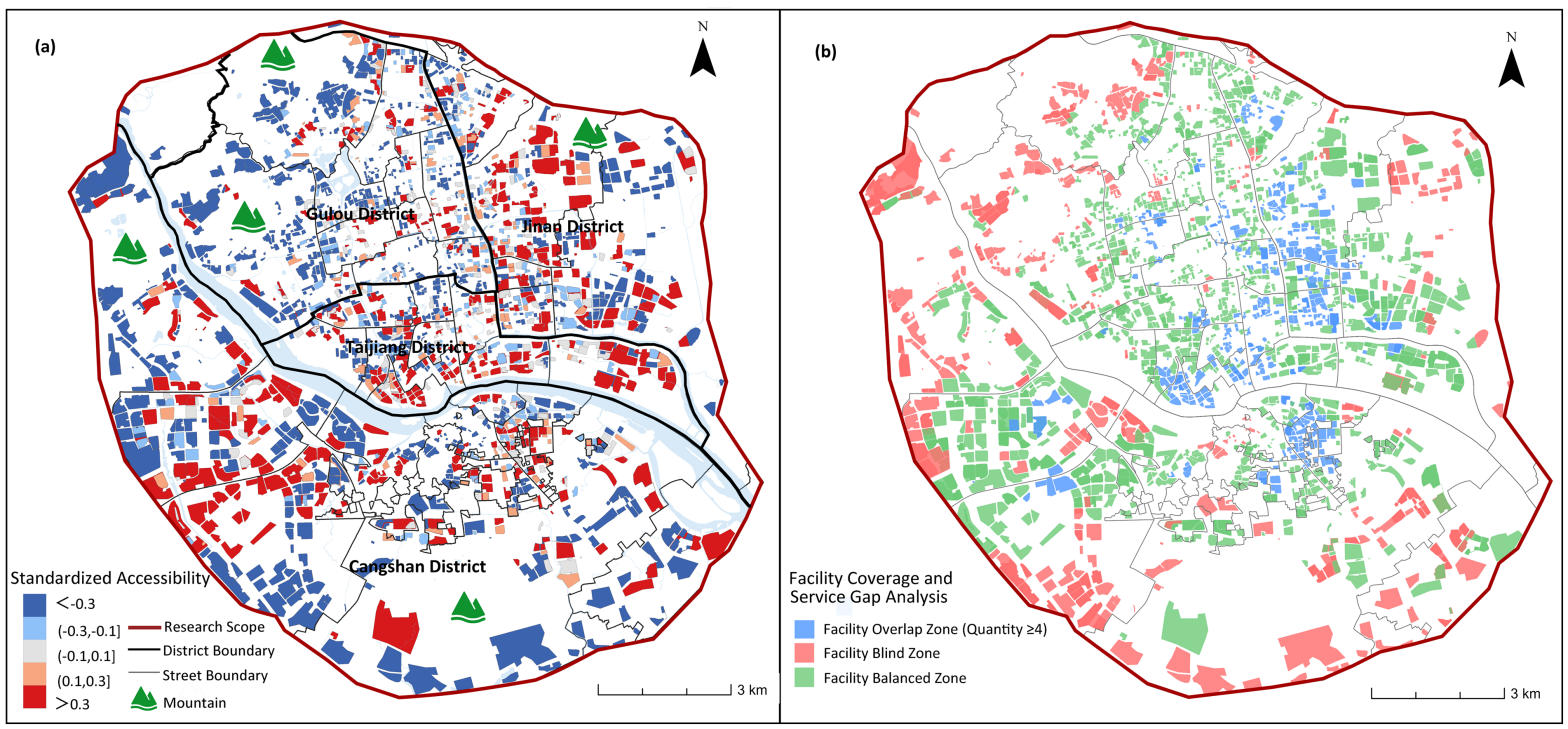
Spatial accessibility of home-based service stations.

In Figure 9, the study sets the accessibility service radius of home-based care service stations at 500 meters. The spatial distribution of high-accessibility areas shows a relatively balanced characteristic. High-accessibility residential areas are more concentrated in the central urban area, showing a gradient decrease outward toward the periphery (see Figure 9). Low-accessibility areas are mostly concentrated in the outer urban areas, and there are obvious service gaps due to topographical conditions and the spatial form of urban villages. From a holistic perspective, the central urban area exhibits an excessive concentration of home-based care service stations, whereas the peripheral regions are characterized by inadequate provision. This imbalance has hindered the realization of the planning objective of achieving balanced coverage across the entire region.

The analysis (see Figure 9b) reveals generally favorable accessibility of home-based care service stations, with service blind zones accounting for only 19.8% of the total area, significantly lower than institutional and community older adults care facilities. However, service redundancy affects 38.3% of the area, far exceeding levels observed in institutional and community care facilities. These over-concentrated configurations predominantly occur in central urban areas, indicating oversupply of home-based care service stations in the urban core.

**Figure 10:**
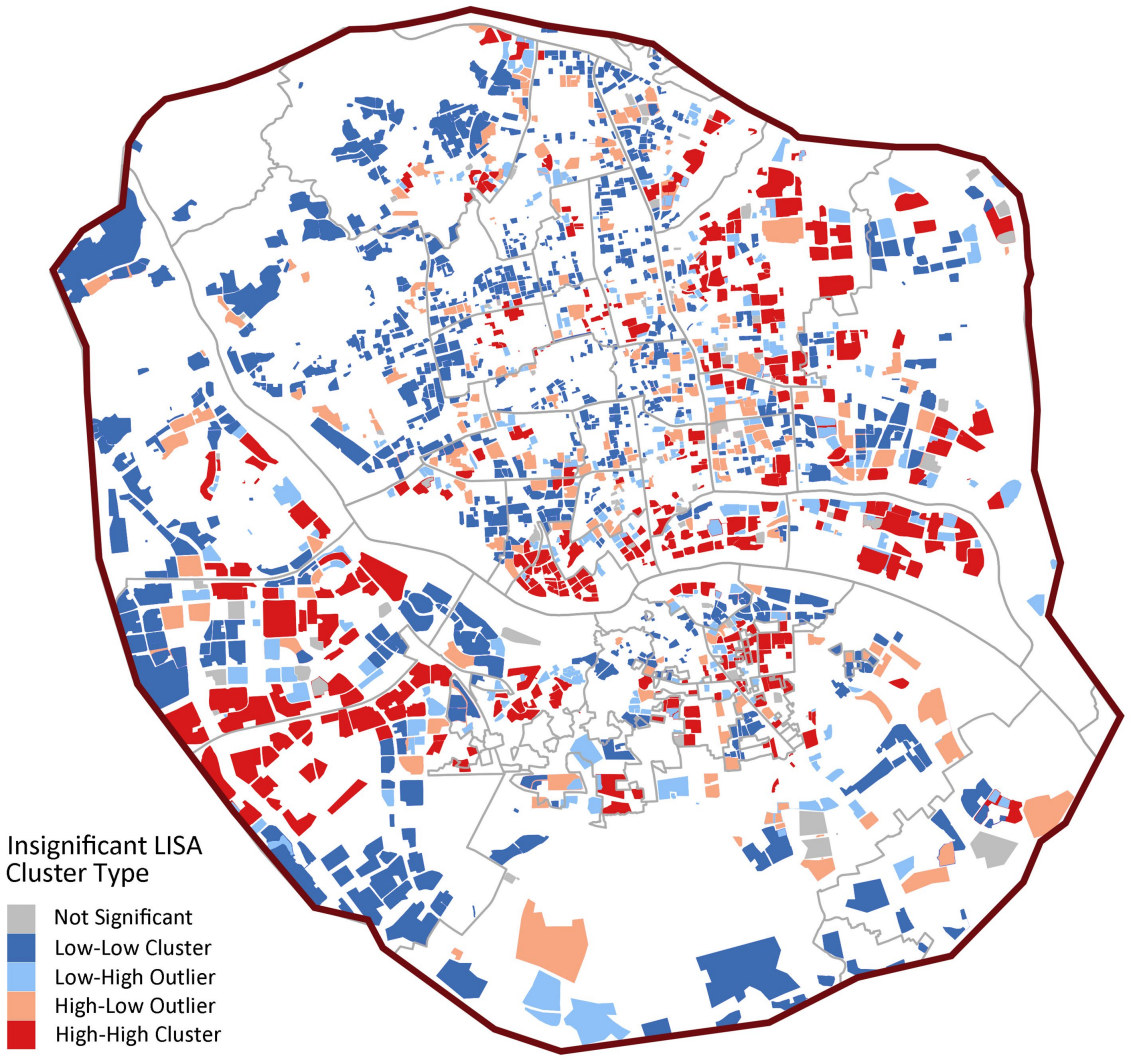
LISA cluster map of home-based care service stations.

In Figure 10, home-based stations exhibit moderate spatial dependence (Global Moran’s *I* = 0.2018, *p* < 0.001) with 49.2% “low-low” clusters (1,120 communities), markedly lower than other facility types. The proportion of spatial outliers is notable (“low-high”: 16.4%; “high-low”: 10.9%), revealing structural issues of coexisting service gaps and redundancies at local scales.

#### 4.1.4 Comparative analysis of three facility types

A comprehensive analysis of the three types of older adults care service facilities, based on Table 3, reveals that among the 2,275 residential communities, only 567 have accessible institutional care service facilities (βi > −0.1), accounting for 24.9% of the total. Similarly, 801 communities (35.3%) have accessible community day care centers, while 844 communities (37.1%) have accessible home-based care service stations.

**Table 3:** Distribution of standardized accessibility levels for three types of older adults care facilities.

Standardized accessibility volume (βi)	Institutional care facilities number (%)	Community day care centers/number (%)	Home-based service stations/number (%)
Significantly low <−0.3	1,186(52.1)	1,220(53.6)	1,117(49.7)
Slightly low (−0.3,−0.1]	522(22.9)	254(11.2)	314(13.8)
Average (−0.1,0.1]	241(10.6)	218(9.6)	220(9.7)
Slightly high (0.1,0.3]	71(3.1)	138(6.1)	167(7.3)
Significantly high >0.3	255(11.2)	445(19.6)	457(20.1)

The table shows the service coverage of three types of older adults care facilities based on the standardized accessibility coefficient (βi). The values are the number of residential areas and their percentages.

Overall, the accessibility of all three facility types remains low. Although home-based care service stations and community day care centers demonstrate relatively higher accessibility than institutional care service facilities, this advantage is not statistically significant. The national “9,073” older adults care service framework, where 90% of seniors receive home-based care, 7% rely on community care, and 3% utilize institutional care positions, home-based care as the system’s cornerstone (29). However, home-based care service stations, serving as the core infrastructure for home-based care, currently exhibit not only significant spatial deficiencies but also fail to provide leading supportive capacity.

**Figure 11:**
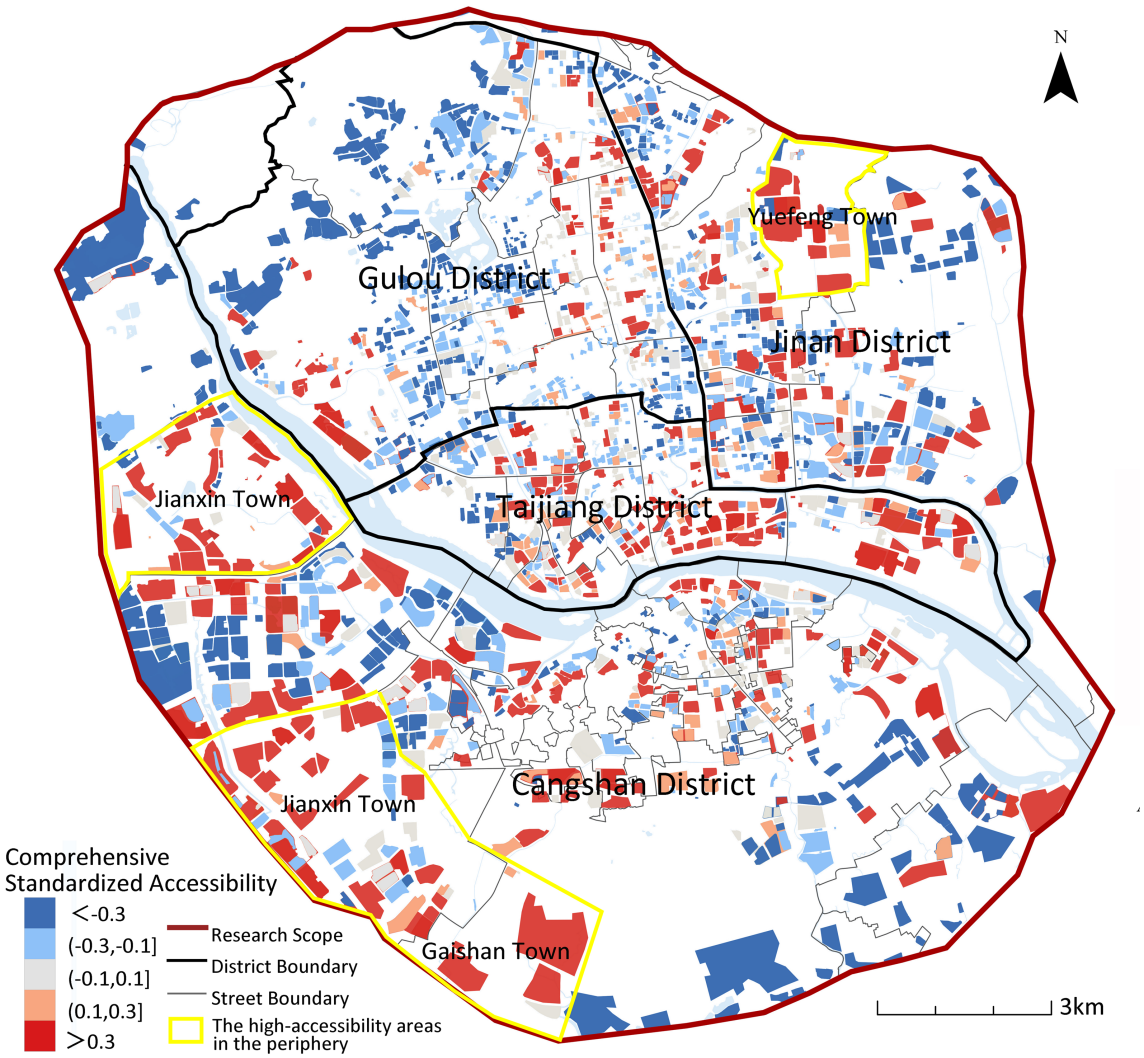
Composite spatial accessibility of institutional/community/home-based older adults care services across Fuzhou’s urban area.

As shown in Figure 11, high-quality senior care resources in Fuzhou are mainly concentrated in the central urban area, forming a large-scale contiguous high-accessibility area, and as the distance from the city center increases, the accessibility level generally decreases. In the peripheral areas, there are some high accessibility areas sporadically distributed, mainly located in the southwestern Jianxin Town area and Yuefeng Town of Jinan District in the northeastern part of Jinan, but in most of the peripheral areas, the accessibility of care facilities for older adults is generally low. This indicates that high-quality senior care resources are still highly concentrated in the central areas, while the sporadic high value areas in the periphery indicate that urban care facilities for older adults have not formed a networked and balanced layout.

### 4.2 Disparities in accessibility across areas and older adults populations

Based on the calculated disparity between willingness-weighted accessibility and comprehensive accessibility of older adults care facilities, this study identifies distinct spatial differentiation patterns between the two metrics (see Table 4).

**Table 4:** Comparative statistics of comprehensive vs. willingness-weighted accessibility metrics.

Accessibility Classification	Mean value	Standard deviation	Minimum value	Maximum value	Median	First quartile	Third quartile
Comprehensive accessibility	0.948	2.305	0	42.547	0.358	0.141	0.792
Willingness-weighted accessibility	0.453	0.866	0	15.317	0.226	0.107	0.453

Questionnaire results reveal that older adults individuals exhibit significantly stronger preference for home-based care service stations compared to community day care centers or institutional care service facilities. Incorporating actual care preference weights into accessibility analysis yields a dual effect: it diminishes the contribution of institutional care service facilities, which typically feature larger service radii and higher accessibility values, while amplifying the weight coefficients of institutional care service facilities and community day care centers (generally characterized by smaller service radius and lower accessibility values).

**Figure 12:**
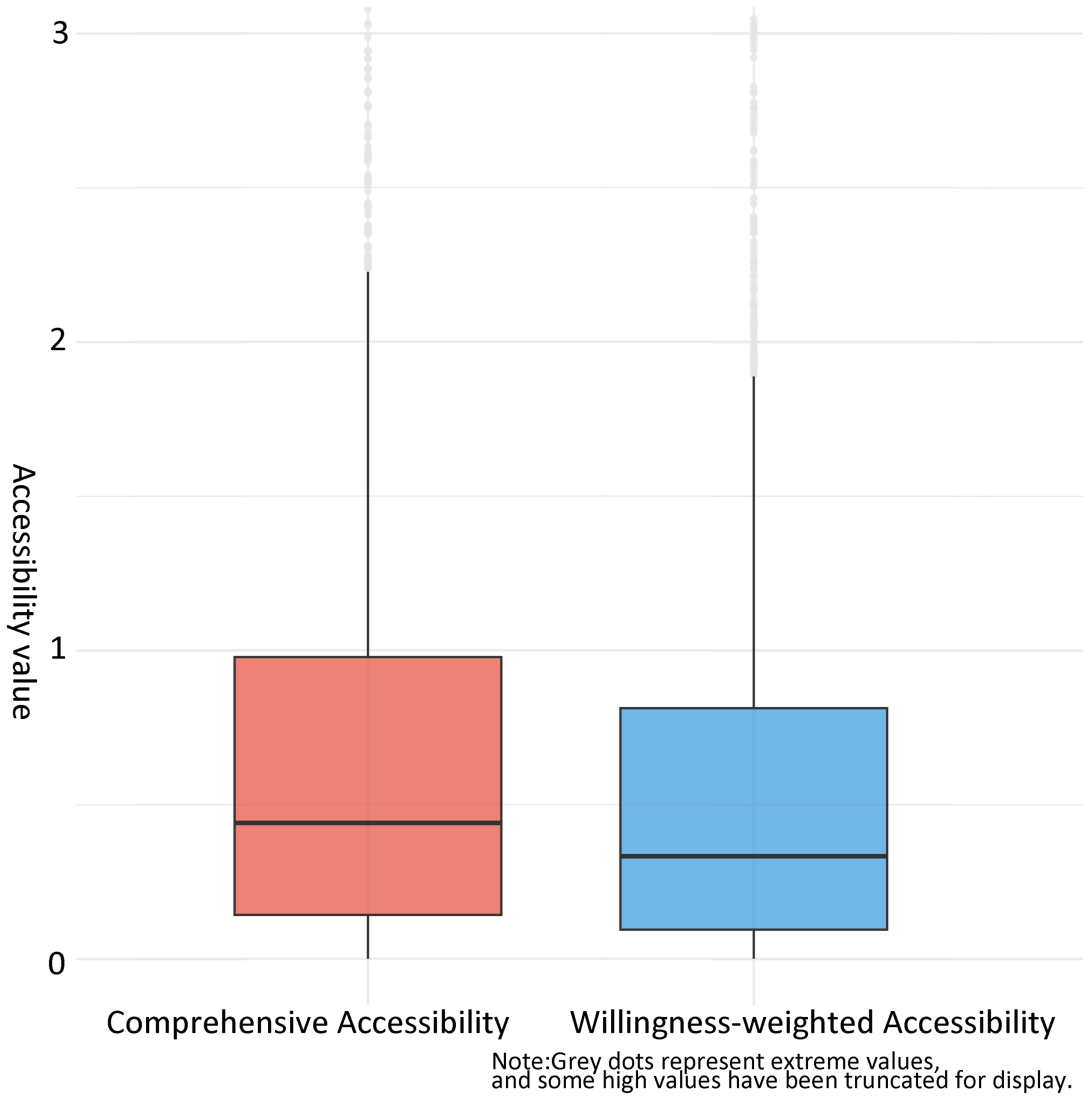
Comparative boxplots of willingness-based and comprehensive accessibility distributions.

Analysis of the distribution characteristics, supported by boxplots (Figure 12) and statistical summary (Table 4), reveals fundamental differences between the two accessibility measures. The distribution of willingness-weighted accessibility shows that most communities are concentrated in the range of 0.107–0.453 (interquartile range), with a median of 0.226 and a standard deviation of 0.866, indicating relatively balanced distribution dominated by medium to low values. In contrast, comprehensive accessibility exhibits characteristics of “high-value agglomeration and extreme differentiation,” with an interquartile range of 0.141–0.792, a median of 0.358, and a substantially higher mean value of 0.948.

To statistically validate these observed differences, a Mann–Whitney U test was conducted after confirming violation of normality assumptions (Shapiro–Wilk test, *p* < 0.05). The test revealed a highly significant difference between the two measures (*W* = 2,097,719, *p* < 0.001), with a median difference of 0.212 (95% CI, 0.197, 0.227), systematically confirming that *β* values were significantly lower than *α* values. This statistical evidence demonstrates that incorporating older adults preferences fundamentally alters accessibility outcomes and provides robust support for the conclusion that the equal-weight comprehensive accessibility metric overestimates service equity relative to the willingness-weighted measure. The pronounced disparity in standard deviations (approximately 2.66 times higher for comprehensive accessibility) further reflects the spatial heterogeneity of facility distribution, where concentrated layout in certain areas substantially elevates local accessibility in the comprehensive model while the willingness-weighted model reveals a more balanced but generally lower accessibility pattern, highlighting gaps in the current layout of home-based care service facilities.

**Figure 13:**
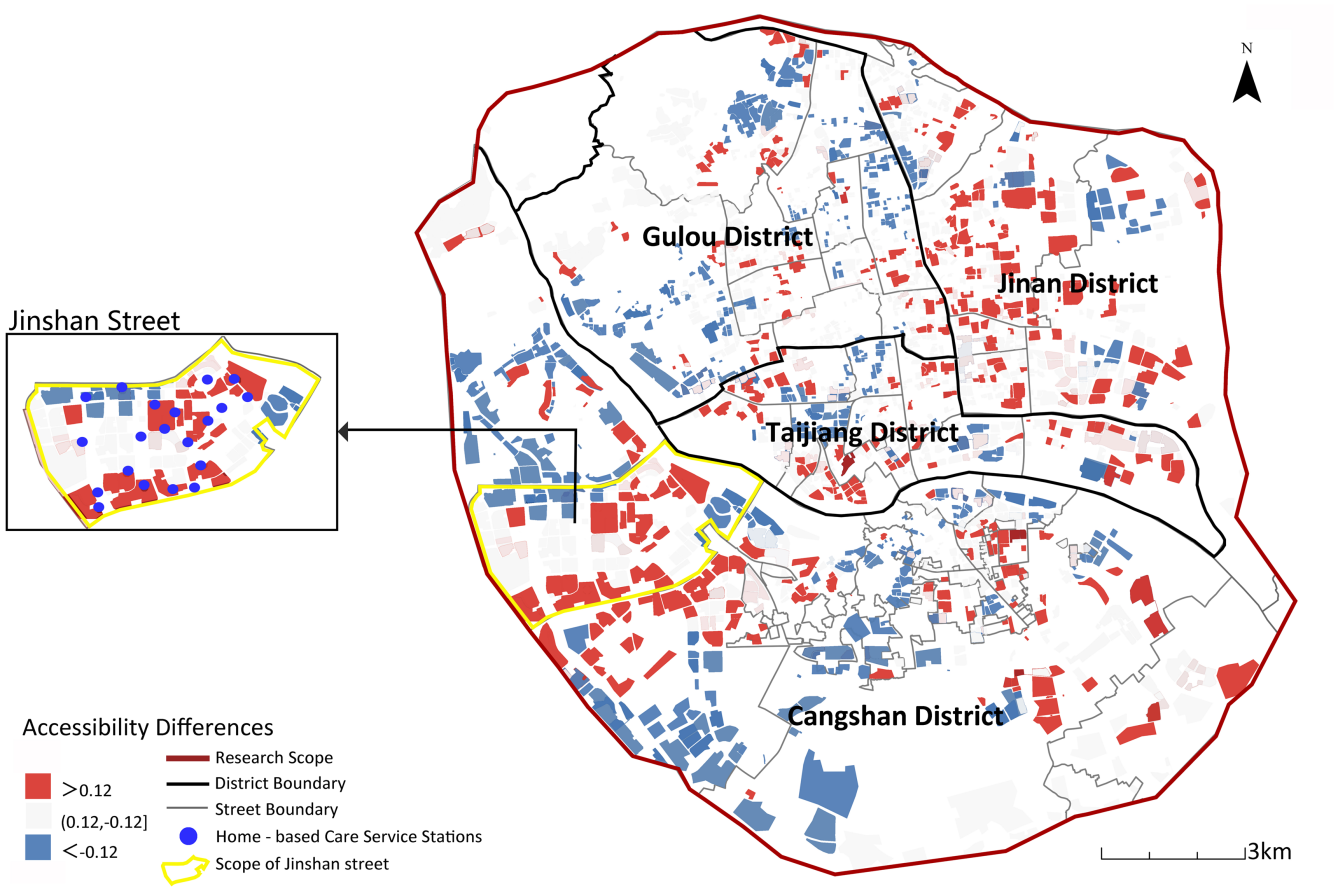
Spatial differences between comprehensive accessibility and willingness-weighted accessibility.

Figure 13 illustrates accessibility changes after incorporating care preferences, revealing significant spatial differentiation. Accessibility declines across most central urban areas while remaining persistently low in peripheral zones. Within central districts (Gulou, Taijiang), facility diversity is counteracted by low weighting coefficients in calculations, reducing overall accessibility metrics. Peripheral areas suffer chronically low accessibility due to facility scarcity. An exception lies within the yellow boundary in Figure 13 highlighting Jinshan Street (Cangshan District), where accessibility improved due to its dense, balanced distribution of home-based care stations (20 of Cangshan’s 92 stations, 21.7%).

Accessibility results incorporating the older adults’ willingness weights indicate a significant mismatch between the spatial allocation of existing older adults care facilities and the actual demand structure. Therefore, taking home-based care service institutions as a starting point to improve accessibility in these areas should be a key priority in future planning.

### 4.3 Optimization strategies for older adults care facility layout based on accessibility analysis

Based on the disparity between willingness-weighted accessibility (*β*) and comprehensive spatial accessibility (*α*), streets are classified into three categories: gain type (β > α), attenuation type (α < β), and risk type (α > β). The disparity change between willingness-weighted accessibility and comprehensive accessibility “+” indicates that willingness-weighted accessibility is improved relative to comprehensive accessibility after incorporating the older adults’ selection willingness, while “−” denotes that willingness-weighted accessibility is reduced relative to comprehensive accessibility. See Table 5 for the spatial distribution and characteristics of these street types.

**Table 5:** Classification of street types based on changes in disparities between willingness-weighted accessibility and comprehensive accessibility.

Street type		Accessibility relationship			Description	Classification
		Comprehensive accessibility (α)	Willingness—based accessibility (*β*)	Disparity change		
Enhancement (β>*α*)	Balanced optimization	High	High	+	The three types of accessibility in this street are all good, which indicates that these service stations are well-constructed and reasonably laid out.	Cangshan District (Sancha Street, Xiadu Street) Taijiang District (Xincha Street, Cangxia Street, Aofeng Street) Gulou District (Dongjie Street)
	Home-care supplement	Low	Low	+	This indicates that although there are home-based care service stations in the street, their quantity is insufficient to support the accessibility of all types of facilities in the entire street.	Cangshan District (Chengmen Town) Taijiang District (Ninghua Street) Jin’an District (Gushan Town, Chayuan Street) Gulou District (Nanjie Street, Shuibu Street)
	Home-care leading	Low	High	+	This indicates that its home-based care service stations are fully equipped, but there is a lack of institutional or community older adults care facilities.	Cangshan District (Jinshan Street) Jin’an District (Yuefeng Town, Wangzhuang Street, Xiangyuan Street)
Decline (α >β)	Facility deficient	Low	Low	−	This indicates that the supply of all three types of care facilities for older adults is insufficient, and these areas are classified as regions with a shortage of care facilities for older adults	Cangshan District (Cangqian, Chating, Luozhou Town) Gulou District (Wufeng Street, Huada, Antai, Hongshan Town, Guxi Street)
	Home-care deficient	High	High	−	This indicates that the institutional or community older adults care facilities in these areas are relatively well-developed, but the allocation of home-based care facilities is insufficient.	Cangshan District (Linjiang Street, Cangshan Town, Duihu Street, Jianxin Town, Gaishan Town) Taijiang District (Shanghai Street, Houzhou, Yangzhou, Yingzhou) Gulou District (Gudong Street)
Risk (α>β)	Structural deficient	High	Low	−	This indicates that there is a problem with the current facility structure: the allocation of home-based care service stations is incomplete, and there is redundancy in community or institutional older adults care facilities.	Taijiang District (Yizhou Street) Gulou District (Wenquan Street)

**Figure 14:**
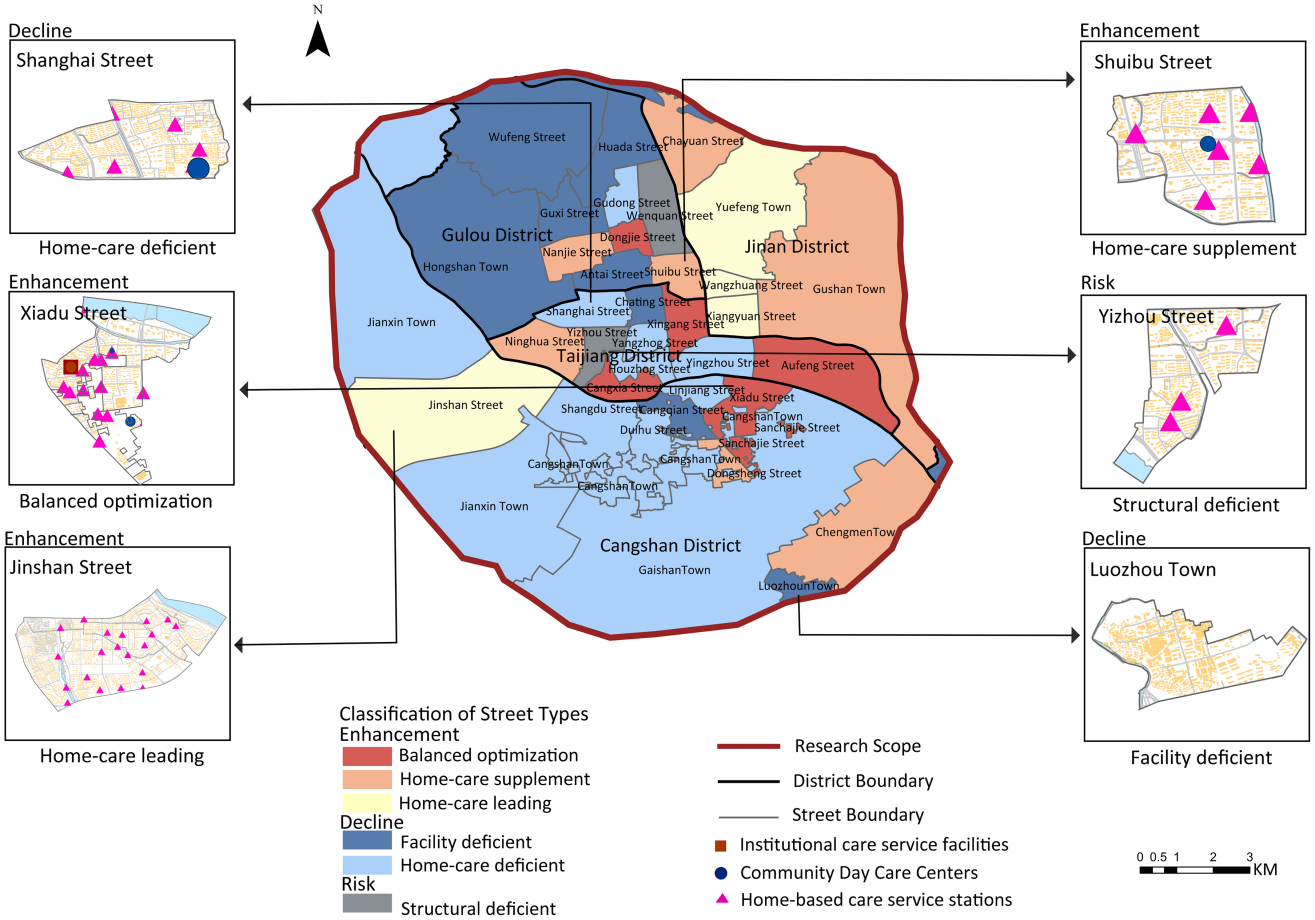
Differentiated service optimization strategies by street classification (gain/attenuation/risk).

**Figure 15:**
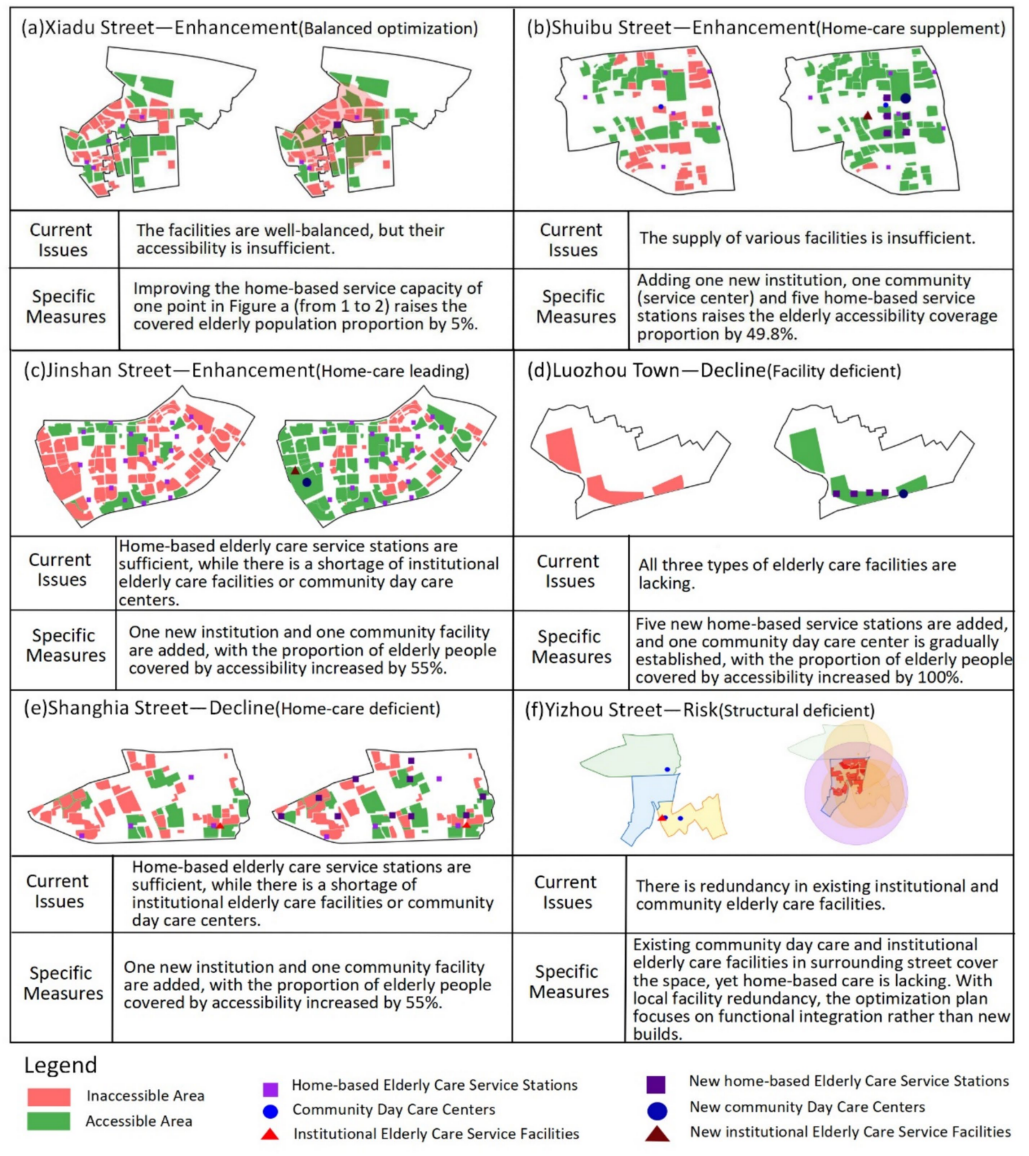
Typology-based optimization strategies for older adults care facility allocation.

Based on the street classification (Gain, Attenuation, Risk) and accessibility characteristics, combined with older adults care preferences and facility supply–demand matching, the following targeted optimization strategies are proposed (Figures 14, 15).

Gain-type streets exhibit high comprehensive accessibility and willingness-weighted accessibility, with a positive disparity (*β* > *α*, “+”). These areas benefit from a balanced mix of institutional care service facilities, community day care centers, and home-based care service stations, with particularly well-developed home-based care service networks. Optimization should therefore prioritize enhancing the quality and standardization of home-based care services, as demonstrated in Xiadu Street (Figure 15a) where doubling the service capacity of one home-based station increased coverage by 5%. However, other gain-type areas (e.g., Shuibu Street) show low overall accessibility despite having all three facility types, necessitating comprehensive expansion. Quantitative simulation for Shuibu Street (Figure 15b) shows that adding one institutional facility, one community center, and five home-based stations increases coverage by 49.8%. Additionally, streets like Jinshan Street (Figure 15c) display adequate home-based station coverage but deficits in institutional or community facilities. Simulation confirms that adding one institutional and one community facility here increases coverage by 55%.

Attenuation-type streets show reduced willingness-weighted accessibility relative to comprehensive accessibility (*α* > *β*, “−”). Among these, areas like Luozhou Town suffer from critically low accessibility levels, reflecting a severe shortage of all three facility types. These areas (Figure 15d) demand urgent investment, with simulation showing that adding five home-based stations and one community day care center can increase coverage by 100%. Conversely, streets such as Shanghai Street (Figure 15e) maintain high baseline accessibility but show significant decrease under preference weighting, revealing adequate institutional/community facilities but insufficient home-based coverage. Optimization here requires focused expansion of home-based stations, with simulation indicating that adding 10 home-based stations increases coverage by 13.3%.

Risk-type streets (defined by α > β and “−”) present high comprehensive accessibility but low willingness-weighted accessibility, indicating structural supply–demand imbalance. In Yizhou Street (Figure 15f), for example, while existing community day care centers and institutional facilities provide spatial coverage, there is a pronounced shortage of home-based care services. Optimization should prioritize functional integration over new construction, as surrounding streets already contain redundant facilities that could serve Yizhou’s population through coordinated planning. For oversupplied home-based stations observed in central urban areas (38.3% redundancy), this study recommends repurposing them into integrated community hubs by incorporating professional services like rehabilitation therapy and day care programs, thus transforming redundant facilities into community assets while addressing specific service gaps.

## 5 Conclusion

This study investigates the supply–demand matching of older adults care facilities in Fuzhou’s main urban area by integrating spatial accessibility analysis with older adults preference weights. This study yields three principal findings. First, significant spatial disparities exist across facility types: institutional care accessibility is generally low and better in outer urban areas, community-based care is concentrated centrally leaving peripheral gaps, and home-based care remains uneven despite broader coverage. Second, integrating older adults preferences reveals a pronounced mismatch: central areas have adequate facilities but of mismatched types, while outer areas suffer from overall undersupply, though Jinshan Street demonstrates how dense, balanced home-based station layout can improve alignment. Third, street-level typology classifies areas into gain-, attenuation-, and risk-type zones, suggesting targeted strategies such as optimizing home-based station distribution in gain-type streets, supplementing missing facilities in attenuation-type areas, and reallocating redundant resources in risk-type zones. These findings offer evidence-based insights for achieving spatially and socially balanced older adults care planning.

This research has several limitations that point toward valuable directions for future research. In terms of data sources, the use of WorldPop raster data, even when calibrated with street-level census information, offers limited granularity in capturing fine-scale variations in older adults population distribution within residential communities. The sampling approach, based on a quota-based convenience method, helped ensure spatial coverage across districts but may not fully represent the preferences and mobility constraints of vulnerable subgroups such as the disabled or empty-nest older adults. This could lead to an overestimation of overall accessibility in the willingness-based model. Similarly, the MG2SFCA method employed fixed walking-distance thresholds, which do not explicitly incorporate multi-modal travel options, terrain barriers, or the spectrum of mobility levels among older adults. These simplifications, while necessary to establish a consistent baseline measurement, may affect the precision of the accessibility estimates. Despite these limitations, the present study offers a valuable and systematic assessment of spatial accessibility inequities and provides an important foundation for understanding supply–demand mismatches in older adults care provision. Future research can constructively build upon this work by integrating real travel-time data derived from multi-modal transport networks, implementing more inclusive sampling strategies to better represent vulnerable subgroups, and developing dynamic, health-sensitive accessibility models. Such advancements would further enhance the equity and practical relevance of planning frameworks for older adults care facility allocation.

## Data Availability

The raw data supporting the conclusions of this article will be made available by the authors, without undue reservation.

## Footnotes

^1^ https://fzsylfw.com/psp/pc/index.html

^2^ https://yanglao.mca.gov.cn/#/elderlyCareMap

^3^ https://www.worldpop.org

^4^ https://code.earthengine.google.com/
